# Dual targeting of IGF-1R and ErbB3 as a potential therapeutic regimen for ovarian cancer

**DOI:** 10.1038/s41598-019-53322-y

**Published:** 2019-11-14

**Authors:** Adam J. Camblin, Gege Tan, Michael D. Curley, Isabel Yannatos, Sergio Iadevaia, Victoria Rimkunas, Mari Mino-Kenudson, Troy Bloom, Birgit Schoeberl, Daryl C. Drummond, Alexey A. Lugovskoy, Chrystal U. Louis, Vasileios Askoxylakis

**Affiliations:** 1 Merrimack Pharmaceuticals, Inc, Cambridge, MA USA; 2 Massachusetts General Hospital, Harvard Medical School, Boston, MA USA

**Keywords:** Ovarian cancer, Cancer therapeutic resistance

## Abstract

Therapeutically targeting receptor tyrosine kinases has proven to be paramount to overcoming chemotherapy resistance in several cancer indications, improving patient outcomes. Insulin-Like Growth Factor Receptor 1 (IGF-1R) and Epidermal Growth Factor Receptor 3 (ErbB3) have been implicated as two such drivers of resistance, however their simultaneous role in ovarian cancer chemotherapy resistance remains poorly elucidated. The aim of this work is to determine the effects of dual IGF-1R/ErbB3 inhibition on ovarian cancer cell signaling, growth, and *in vivo* efficacy. Assessment of *in vitro* chemotherapy response across a panel of ovarian cancer cell lines revealed that increased IGF-1R cell surface expression correlates with decreased sensitivity to chemotherapy, and that growth induced by IGF-1R and ErbB3 ligands is blocked by the tetravalent bispecific antibody targeting IGF-1R and ErbB3, istiratumab. *In vitro* chemotherapy treatment increased ovarian cancer cell line capacity to activate prosurvival PI3K signaling in response to ligand, which could be prevented with istiratumab treatment. Furthermore, *in vivo* efficacy of standard of care chemotherapies using a xenograft model of ovarian cancer was potentiated with istiratumab. Our results suggest a role for IGF-1R and ErbB3 in driving chemotherapy resistance of ovarian cancer.

## Introduction

Ovarian cancer is the fifth most common malignancy in women representing a leading cause of cancer-related death^1^. In 2018 more than 22,000 women in the USA were diagnosed with ovarian cancer, with 14,000 ovarian-cancer related deaths^2^. Due to late onset of symptoms more than two thirds of patients are diagnosed with locally advanced or metastatic stage disease^2^, and established therapeutic regimens include combinations of platinum- and taxane-based chemotherapies (NCCN Guidelines). Initial treatment responses are common, however, they are usually followed by disease recurrence. This underscores the urgent need for a deeper understanding of ovarian cancer pathophysiology and the development of novel therapeutic approaches.

Receptor tyrosine kinases may have a critical role in the pathophysiology of ovarian cancer. Among these, insulin growth factor 1 receptor (IGF-1R) is implicated in development, progression, metastasis and chemotherapy resistance of ovarian cancer^3^. IGF-1R, its activating ligands (IGF-1, IGF-2), as well as regulating insulin growth factor binding proteins (IGFBP), are expressed in ovarian malignancies, supporting the hypothesis that IGF-1R signaling might be a promising therapeutic target^4,5^. As a result, strategies to inhibit IGF-1R signaling using blocking antibodies or small molecule signaling inhibitors have been developed and tested in ovarian cancer. While IGF-1R targeted approaches showed promising preclinical activity, they have thus far failed to provide clinical benefit^3,6^.

The complexity of IGF-1R signaling in ovarian cancer requires a deeper understanding and knowledge of the pathway in the disease. IGF-1R can interact and heterodimerize with other receptor tyrosine kinases (RTKs), which may alter ligand affinity and activation of downstream signaling^3^. Moreover, upon blocking of the IGF-1R pathway, other RTKs may compensate by re-activation of pro-survival PI3K/AKT signaling. Increasing evidence in recent years suggests a distinct role for ErbB3 signaling in ovarian cancer. An autocrine signal-transducing loop involving ErbB3 and its activating ligand HRG has been found to promote cell proliferation in human ovarian cancer cells and the effects of HRG/ErbB3 were abrogated by genetic or pharmacological ErbB3 inhibition^7^. Moreover, tissue analysis from ovarian cancer patients indicated an association of ErbB3 or HRG expression with decreased patient survival^8,9^. Further studies have revealed a high prevalence of ErbB3 and its ligand HRG in ovarian cancer^10,11^.

In this study we characterized the impact of IGF-1R and ErbB3 in ovarian cancer growth and therapy resistance and reveal strategies to re-sensitize ovarian cancer cells to clinically relevant chemotherapy using istiratumab, a fully human bispecific tetravalent IGF-1R- and ErbB3-targeting antibody, composed of a monoclonal IgG1 antibody, engineered to contain two single-chain Fv fragments^12,13^. Istiratumab has been previously shown to have two modes of action: (i) it blocks IGF-1, IGF2, and HRG binding to their receptors; and (ii) it induces degradation of receptor complexes containing IGF-1R and ErbB3^13^. Our data indicate a potential benefit of dual IGF-1R/ErbB3 inhibition for ovarian cancer treatment, and highlight the potential impact of istiratumab in combination with standard of care chemotherapy to treat ovarian cancer.

## Results

### Sensitivity to chemotherapy in ovarian cancer cell lines correlates with cell-surface IGF-1R expression, but not ErbB3 expression

**Figure 1:**
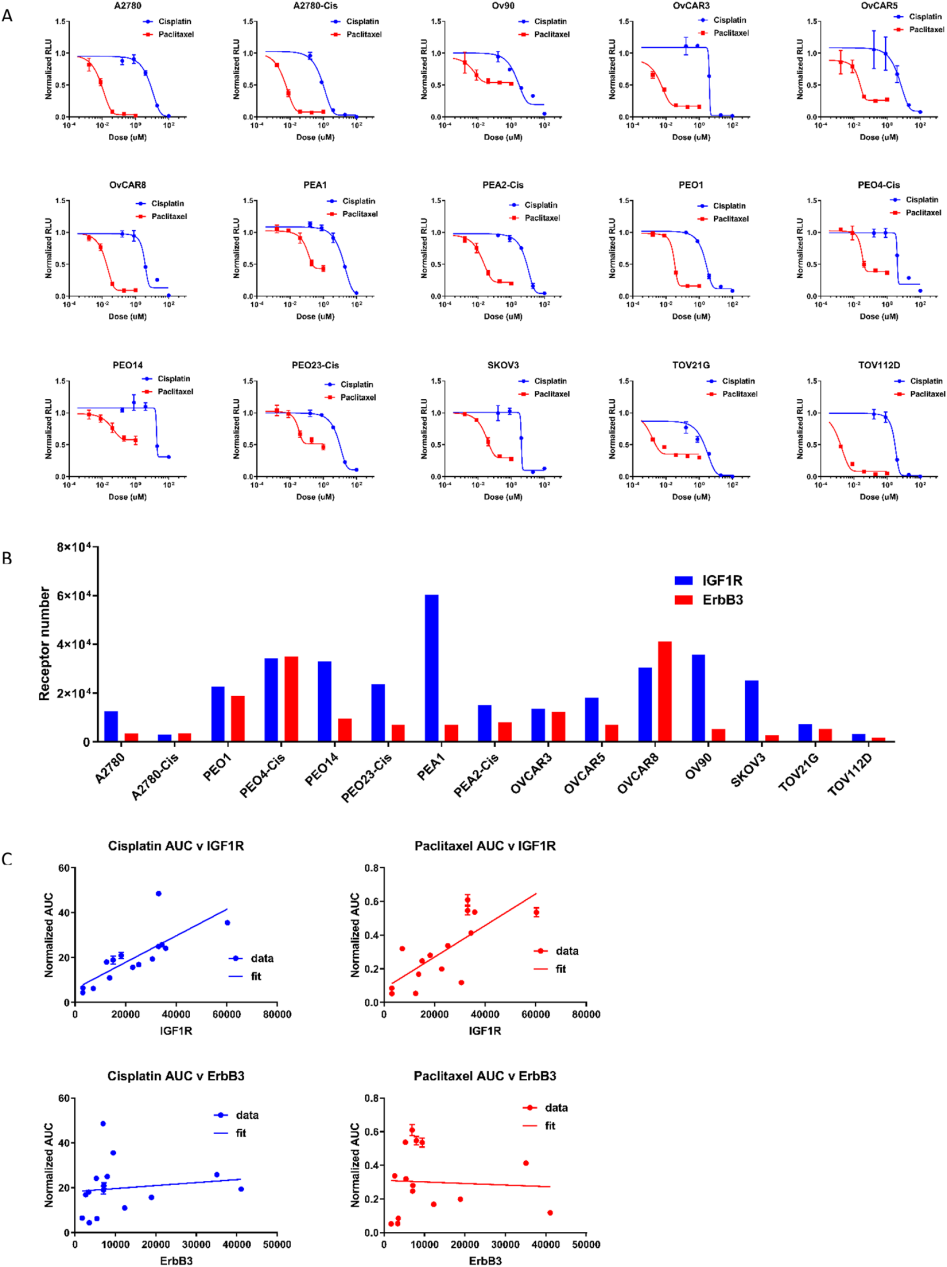
IGF-1R expression correlated with reduced sensitivity to chemotherapy in ovarian cancer cell lines. (**A**) Fifteen ovarian cancer cell lines were treated in triplicate with paclitaxel or cisplatin as indicated for 72 hours, and raw luminescent values were normalized to vehicle control cells. (**B**) Cell surface expression of IGF-1R and ErbB3 were assessed via quantitative flow cytometry across the ten ovarian cancer cell lines. (**C**) Cell line responsiveness to paclitaxel or cisplatin from A correlated to cell surface receptor expression from (**B**).

To investigate the relative importance of cell-surface receptor tyrosine kinase expression to chemotherapy sensitivity in ovarian cancer, we determined the proliferation response to cisplatin or paclitaxel (Fig. 1A) across a panel of fifteen ovarian cancer cell lines. Cell-surface expression of IGF-1R and ErbB3 was determined by quantitative flow cytometry (Fig. 1B). Area under the curve (AUC) of the chemotherapy dose response from Fig. 1A was plotted against receptor expression from Fig. 1B, and the correlation between chemotherapy resistance and RTK expression was determined. These studies identified a statistically significant (p < 0.0006 for cisplatin, p < 0.0014 for paclitaxel) positive correlation between chemotherapy resistance and expression levels for IGF-1R, but not for ErbB3 (Fig. 1C). Investigation of other RTKs revealed a positive correlation between AUC and cMET expression, but not EGFR or HER2 (Supplementary Fig. 1A). A multidimensional partial least squares regression analysis of all receptor expression correlations with *in vitro* chemotherapy sensitivity revealed IGF-1R expression to be the most heavily weighted contributor (Supplementary Fig. 1B), followed closely by cMET expression. 11 cell lines were also profiled for growth factor mRNA expression (Fig. S3), revealing 7/11 may express autocrine HRG, whereas only 3/11 may express autocrine IGF-1.

### IGF-1R, ErbB3 and their ligands IGF-1, IGF2 and HRG are highly expressed in ovarian cancer patient samples

**Figure 2:**
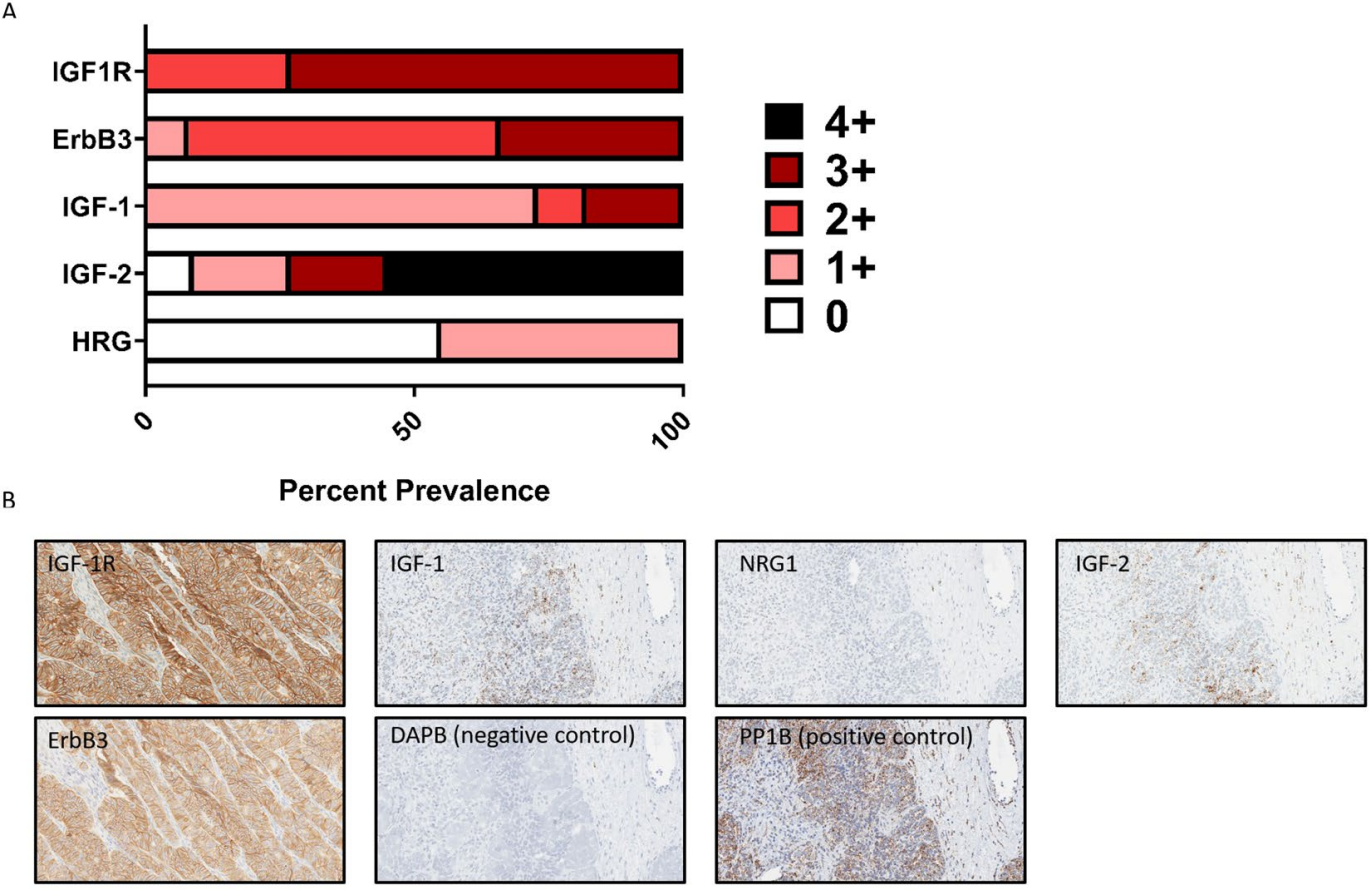
IGF-1R, ErbB3, and their associated ligands are expressed in ovarian tumor patient samples. (**A**) Percent prevalence for IGF-1R, ErbB3, IGF-1, IGF-2, and HRG in ovarian tumor samples as defined as a score between 0 and 3+ for IHC (IGF-1R and ErbB3) or between 0 and 4+ (IGF-1, IGF-2, HRG). (**B**) Representative images from A showing positive staining. Note the diffuse brown staining for IGF-1R and ErbB3 protein staining and the punctate brown dots for IGF-1, IGF-2, and HRG RNA *in situ* hybridization.

A survey of ovarian cancer patient samples was performed for IGF-1R, ErbB3, and their respective ligands. All patient samples tested were positive for IGF-1R protein expression (n = 11) with 27% 2+ and 73% 3+. In regard to ErbB3 expression 58% of patient samples tested were 2+ positive, and 33% were 3+ (n = 12). All samples tested were positive for IGF-1 mRNA expression (n = 11) with 9% 2+ and 18% 3+. Similarly, 91% of patient samples were positive for IGF2 mRNA expression with 18% scoring 3+ and 55% scoring 4+. 55% of patient samples were positive for HRG mRNA expression with a score of 1+ (Fig. 2A, Table S1). Representative images of staining are shown in Fig. 2B.

### Ovarian cancer cell lines proliferate in response to IGF-1 or HRG stimulation *in vitro*

**Figure 3:**
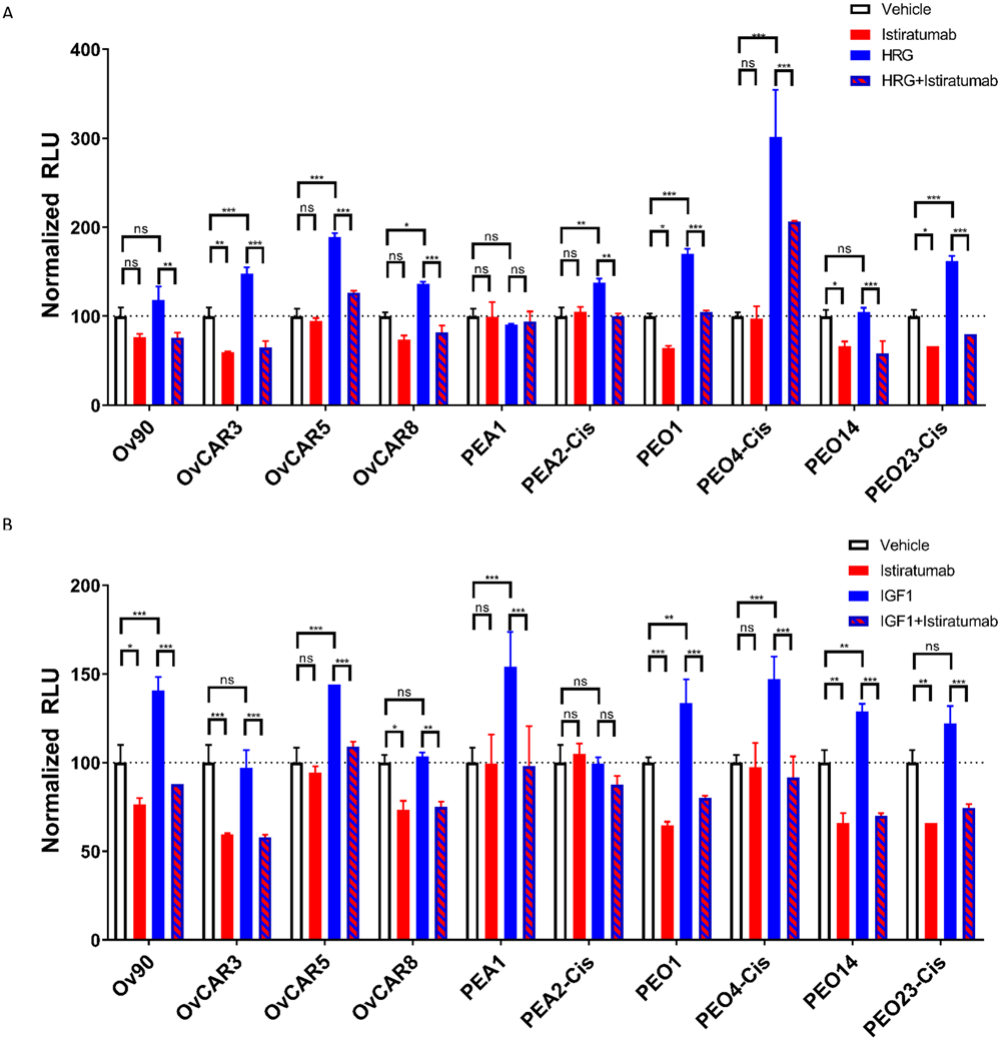
Istiratumab inhibits ligand driven proliferation of ovarian cancer cell lines. Ovarian cancer cells grown overnight in 4% FBS were treated with 50 nM IGF-1 (**A**) 10 nM HRG (**B**) with and without 1uM istiratumab in duplicate as indicated for 72 hours and proliferation was assessed via Cell Titer-Glo® Luminescent Cell Viability Assay. Significance was determined by two-way ANOVA using GraphPad Prism software (*p < 0.033, **p < 0.002, ***p < 0.001).

A panel of ten ovarian cancer cell lines was stimulated with IGF-1 (Fig. 3A) or HRG (Fig. 3B) and proliferation was assessed via Cell Titer-Glo® Luminescent Cell Viability assay after 72 hours. Six out of ten cell lines showed significantly increased proliferation in response to IGF-1 treatment, and seven out of ten showed significantly increased proliferation in response to HRG stimulation. In all but one cell line (PEA2-cis), co-treatment with the bispecific and tetravalent IGF-1R/ErbB3 IgG istiratumab resulted in reduction in cancer cell proliferation compared to untreated cells in the presence of IGF-1 (Fig. 3A), whereas in all but one cell line (PEA1) a decrease in proliferation was noted compared to untreated cells in the presence of HRG (Fig. 3B). Of note, dual IGF-1R/ErbB3 inhibition significantly decreased cell proliferation in six cell lines even in the absence of recombinant IGF-1 (Fig. 3A), whereas this was the case for four cell lines in the absence of recombinant HRG (Fig. 3B). Interestingly, no statistically significant correlation between receptor IGF-1R or ErbB3 (r^2^ = 0.008965, p = 0.7947 and r^2^ = 0.3197, p = 0.0885 respectively) expression levels and sensitivity to istiratumab was observed (Fig. S4)

### Dual IGF-1R/ErbB3 inhibition blocks IGF-1 and HRG mediated activation of PI3K and MAPK signaling cascades in ovarian cancer cell lines *in vitro*

**Figure 4:**
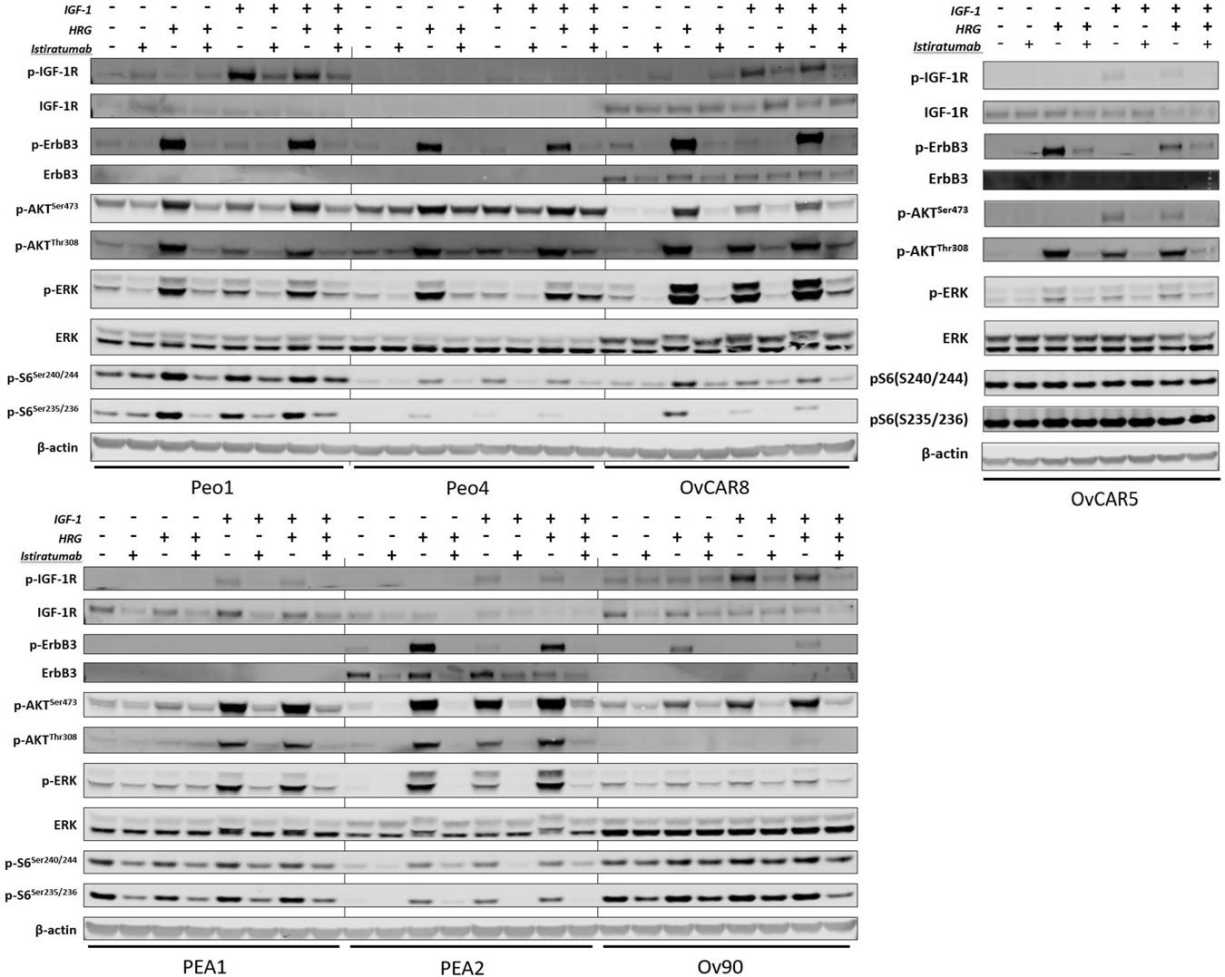
Istiratmab inhibits basal and ligand-induced IGF-1R and ErbB3 signaling in ovarian cancer cell lines. Cells were treated with 1uM istiratumab for 60 min followed by 50 nM IGF-1 and/or 10 nM HRG for 15 min, then lysed and assayed via western blot for PI3K and MAPK signaling activation. Following electrophoresis and transfer of protein samples to nitrocellulose membranes, membranes were cut horizontally into thin slices at the predicted molecular weights of each target protein to allow for blotting of multiple target proteins from a single gel, thus increasing the number of proteins which can be assessed from a single sample without stripping the membrane. Each slice was incubated with the appropriate primary antibody followed by fluorescently labeled secondary antibodies, before being imaged as described in the methods section. Each box presented within the figure shows the entirety of a membrane slice, with each separate slice being delineated by white space surrounding the image. For ease of interpretation, a black vertical line behind the slices delineates when different cell lines were loaded on the same gel, and different treatments are indicated with a “+” symbol.

Seven serum starved ovarian cancer cell lines were stimulated with IGF-1 or HRG for 15 minutes, and then downstream signaling cascades were assessed via western blot. AKT and/or ERK phosphorylation was induced in response to IGF-1 and/or HRG in all seven cell lines (Fig. 4). The addition of the dual IGF-1R/ErbB3 inhibitor istiratumab was able to block activation of the PI3K and MAPK signaling cascades (Fig. 4). Looking at the upstream receptor tyrosine kinases involved, istiratumab treatment inhibited IGF-1R and/or ErbB3 phosphorylation in response to IGF-1 and HRG stimulation. Our data indicate that treatment with istiratumab led to a decrease in total IGF-1R and/or ErbB3 protein levels in some cell lines (Fig. 4). Interestingly cell lines showing the greatest inhibition of proliferation in response to istiratumab monotherapy in Fig. 3 (PEO1, OVCAR8, and OV90) also showed the strongest basal p-IGF1R, suggesting a connection between basal activation levels of this receptor with drug efficacy.

### Chemotherapy potentiates ligand-mediated activation of pro-survival signaling *in vitro*

**Figure 5:**
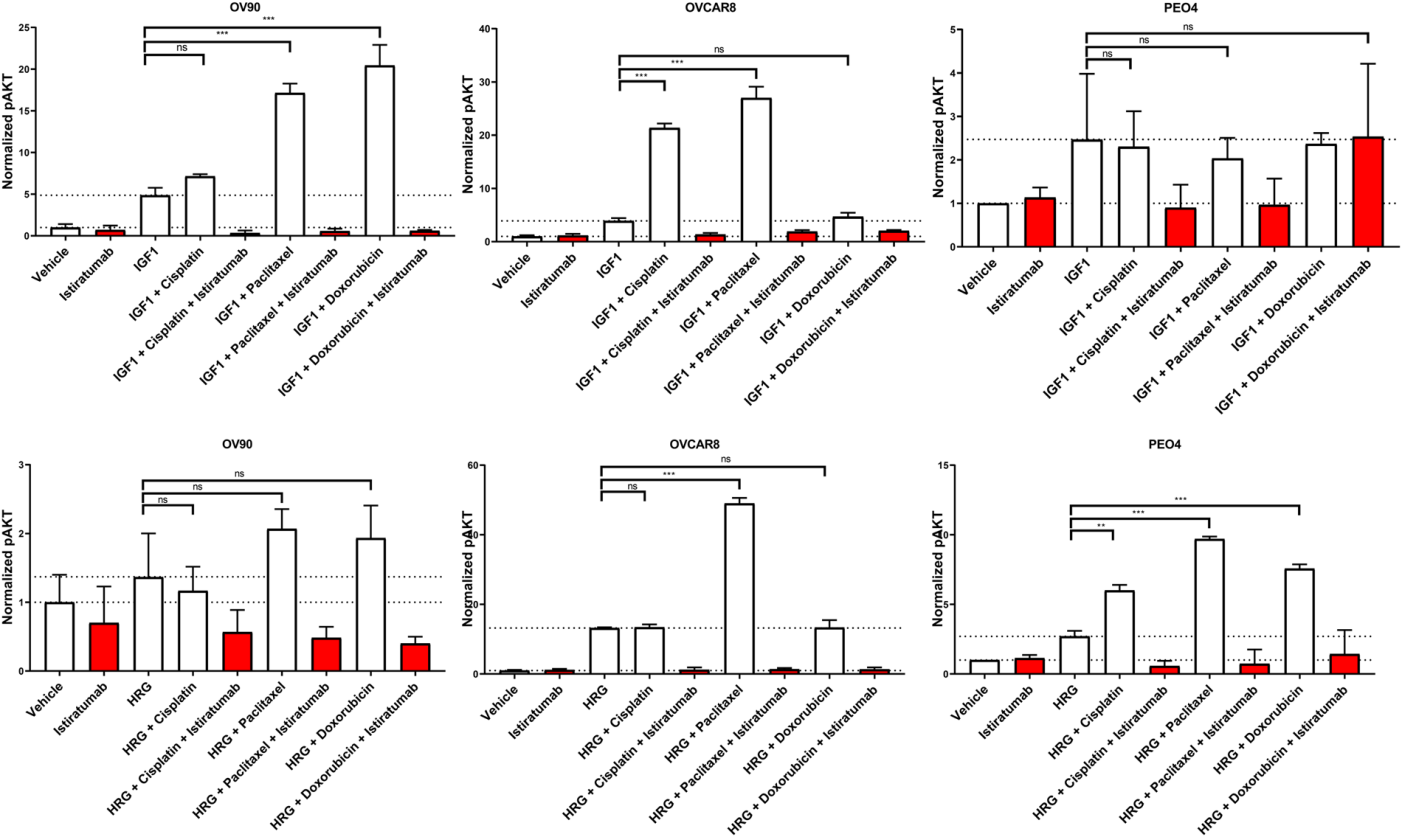
Istiratumab inhibits AKT activation potentiated by chemotherapy and ligand co-treatment. Cells were treated in triplicate as indicated with IGF-1 (**A**) or HRG (**B**) alone or in combination with istiratumab, cisplatin, paclitaxel, or doxorubicin for 24 hours, then lysed and analyzed for AKT Ser473 phosphorylation by ELISA.

To assess how cell lines respond to IGF-1 and HRG while treated with cisplatin, paclitaxel, or doxorubicin, OV90, OVCAR8 and PEO4 cells were cultured overnight with chemotherapies followed by 15 min IGF-1 (Fig. 5A) or HRG (Fig. 5B) stimulation. Cells were lysed and AKT phosphorylation was assessed by ELISA. Consistent with the Western blot analysis (Fig. 4), IGF increased AKT phosphorylation in OV90, OVCAR8, and PEO4 cells (Fig. 5A), whereas HRG enhanced AKT phosphorylation in OVCAR8 and PEO4 cells (Fig. 5B). Of note, chemotherapy treatment further potentiated a striking increase of pAKT in the presence of the growth factors in 9 of 18 chemotherapy-ligand combination conditions, an effect that was strongest for paclitaxel (Fig. 5A,B). In all three cell lines dual inhibition of IGF-1R and ErbB3 with istiratumab resulted in pAKT decrease to background levels (Fig. 5A,B).Of note, chemotherapy alone did not increase AKT phosphorylation levels with statistical significance (Fig. S5).

### Dual IGF-1R/ErbB3 blocking inhibits tumor growth and enhances antitumor activity of chemotherapy *in vivo*

**Figure 6:**
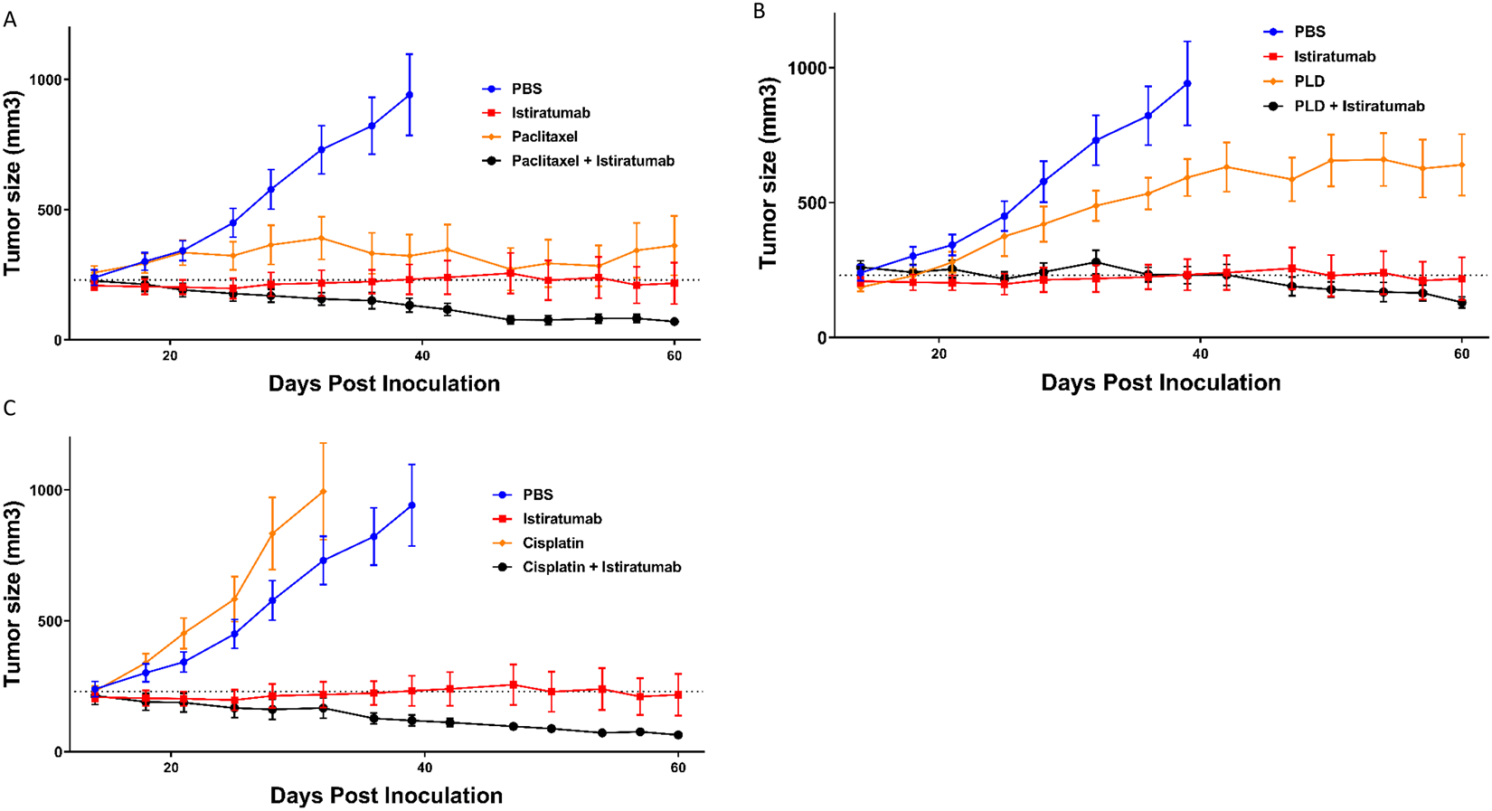
Istiratumab combines with chemotherapy to regress tumor volume in *in vivo* model of ovarian cancer. Mice baring OV90 tumors were segregated in to groups of equal average volume (n = 10) and treated with saline vehicle, 30 mg/kg istiratumab intraperitonially every three days, 20 mg/kg paclitaxel intraperitonially every 7 days (**A**) 5 mg/kg cisplatin intraperitonially every 7 days (**B**) 3 mg/kg pegylated liposomal doxorubicin intravenously every 3 days (**C**) or the combination. Tumor volumes were measured twice weekly. The same control mice and isitratumab treated mice are represented in each of the subfigures for clarity.

We tested the *in vivo* effects of dual IGF-1R/ErbB3 inhibition on the antitumor activity of three different clinically relevant chemotherapies in mice bearing subcutaneous OV90 tumors. The OV90 model was chosen because it grows well *in vivo* and showed sensitivity to the different chemotherapies. Whereas paclitaxel led to tumor control (Fig. 6A) and liposomal doxorubicin significantly delayed tumor growth compared to control (Fig. 6B), OV90 tumors were resistant to cisplatin *in vivo* (Fig. 6C). Istiratumab monotherapy led to tumor stasis, whereas the combination of dual IGF-1R/ErbB3 inhibition with chemotherapy resulted in tumor regression for all three combinations (Fig. 6).

## Discussion

High recurrence rates following chemotherapy in patients with metastatic ovarian cancer emphasize the need for novel treatments. Receptor tyrosine kinases (RTKs) are critical effectors of ovarian cancer progression and therapy resistance. Here we show that IGF-1R, ErbB3 and their ligands are expressed in a significant proportion of ovarian cancer patient samples. A correlation of increasing IGF-1R expression with decreasing sensitivity to cisplatin and paclitaxel was shown across a panel of cell lines representing different subtypes of ovarian cancer, including adenocarcinoma (A2780, A2780Cis, OVCAR8, OVCAR5, PEO14, PEO23, PEA1, PEA2, SKOV3), serous (OVCAR3, OV90, PEO1, PEO4), clear cell (TOV-21G), and endometrioid (TOV-112D) ovarian cancer subtypes, suggesting a broad applicability of this research in this disease indication. Activating ligands of both IGF-1R and ErbB3 promote ovarian cancer cell proliferation and pro-survival signaling activation, whereas dual blocking of IGF-1R and ErbB3 enhances the efficacy of relevant chemotherapies.

IGF-1R is a robustly expressed RTK in ovarian cancer, and high expression of IGF-1R has been previously shown to correlate with resistance to cisplatin chemotherapy^14^. IGF-1R knockdown or signaling inhibition can inhibit ovarian cancer cell proliferation^15,16^ and sensitize cancer cells to platinum-based chemotherapy^17^. Despite the promising preclinical reports, clinical trials did not provide meaningful benefit for ovarian cancer patients treated with IGF-1R inhibitors. This was the case when IGF-1R inhibitors were used as monotherapy after chemotherapy^18^ and when combined with other targeted therapies^19^. The failure of IGF-1R inhibitors in clinical trials is largely attributed to the complexity of RTK pathway signaling, including compensatory pathway activation through other RTKs. Monospecific targeted therapies may be narrowly focused and parallel signaling pathways may promote cancer cell escape from treatment.

IGF-1R can interact with multiple RTKs that are overexpressed in ovarian cancer, such as EGFR, HER2, ErbB3 and c-Met^3^. Our study focused on the interaction between IGF-1R and ErbB3 based on increasing evidence for a critical role of the ErbB3/HRG signaling axis in this disease. ErbB3 belongs to the ErbB family of cell surface receptor proteins, however, in contrast to other ErbB-family members it lacks a functioning kinase domain^20^. Upon binding of the ligand heregulin (HRG), ErbB3 heterodimerization with other RTKs is promoted, resulting in potent activation of pro-survival PI3K/AKT signaling and resistance against various therapeutic modalities, including cytotoxic chemotherapies^12,21^. Screening in ovarian cancer cells using lentivirally-delivered short hairpin RNA library targeting RTKs revealed ErbB3 as a relevant target and both genetic and pharmacological inhibition of the HRG/ErbB3 axis activation resulted in tumor growth inhibition^7,22^.

ErbB3 activation involves protein overexpression through increased *ERBB3* transcription and activation through increased autocrine or paracrine HRG signaling, as well as ligand-independent activation by other RTKs^21,23,24^ or through mutations in the extracellular domain of the protein^25^. Although multiple RTKs are overexpressed in ovarian cancer compared to normal ovarian tissue^8,26–29^, no specific addicting oncoprotein has been clearly validated in the disease and thus the relative impact of different RTKs remains largely unknown. Our analysis of correlation between RTK expression and chemotherapy sensitivity in multiple ovarian cancer cell lines suggested a correlation for IGF-1R and cMET expression, but not ErbB3. This is consistent with previous findings indicating that overexpression of ErbB3 may not be sufficient to promote cancer cell proliferation and survival in isolation, but requires activation by HRG. Depletion of the activating ligand elicited proliferation arrest in ErbB3 activated ovarian cancer cell lines, whereas cell lines with high ErbB3 expression levels but no expression of the activating ligand did not show ErbB3 activation and were not sensitive to ErbB3 targeting^7^. Thus, although our expression analysis in human samples suggests strong ErbB3 expression in a significant proportion of ovarian cancers, a result that is also supported by previous findings^29^, ErbB3 expression alone may not represent a sufficient biomarker for treatment with anti-ErbB3 molecules. We hypothesize that the ErbB3 ligand heregulin (HRG) may have an important role and needs to be critically investigated. Retrospective univariate biomarker analysis from a randomized phase 2 trial of the fully-human monoclonal, ligand competitive anti-ErbB3 agent seribantumab in combination with paclitaxel in patients with advanced platinum-resistant or refractory ovarian cancer suggested that patients with detectable levels of HRG had a progression-free survival hazard ratio that favored the experimental arm, whereas undetectable HRG favored the control arm^9^. Further evidence shows that rearrangements in the gene encoding for HRG, *NRG1*, can drive cancer growth and early clinical data supports the use of ErbB3 targeting approaches in patients with *NRG1*-rearranged malignancies^30^. Together, these findings underscore the necessity to further investigate and unravel the impact of HRG in malignancies and critically consider it in the design of clinical trials targeting ErbB3. Based on recent evidence showing that the tumor microenvironment can cause de novo resistance to PI3K signaling inhibitors by activating the HRG/ErbB3 axis^31^, it is critical that further scientific efforts include investigation of the role of the tumor microenvironment in HRG/ErbB3 mediated effects in cancers, including ovarian cancers.

The close interaction between IGF-1R and ErbB3 signaling has been reported in various malignancies. Mechanistic studies showed that *CD74-NRG1* fusion gene promotes activation of NF-kB signaling pathway in tumors, which in turn enhances the secretion of insulin-like growth factor 2 (IGF-2) and phosphorylation of its receptor IGF-1R^32^. Furthermore, recent data suggest that *NRG* stimulates the transcription of *IGF2* mRNA in an NF-kB-dependent manner^33^. NF-kB binding site motifs have been identified in the promoter sequence of IGF-2, leading to increased IGF-2 production at the transcriptional level. The IGF-1R/ErbB3 interaction is also underscored by data showing that ErbB3 upregulation may compensate for IGF-1R receptor blockade in malignancies and vice versa^12,13,34^. Moreover, chemotherapies have been shown to increase expression of both IGF-1R and ErbB3 in tumor cell lines, rendering these resistant to cytotoxic therapies^12^. Together, these data point to ErbB3 as potentially critical RTK in the treatment of IGF-1R positive tumors, such as ovarian cancer.

Previous preclinical studies showed very promising potential of istiratumab in pancreatic cancer models^12^. These data formed the basis for the CARRIE study, a double-blind placebo-controlled phase II study of istiratumab in combination with nab-paclitaxel plus gemcitabine versus nab-paclitaxel and gemcitabine alone in front-line metastatic pancreatic cancer patients with high free IGF-1 serum levels (www. clinicaltrials.gov; ID: NCT02399137). Despite the promising preclinical activity, istiratumab failed to improve the efficacy of standard-of-care chemotherapy in the front-line treatment of patients with metastatic pancreatic cancer^35^. The reasons for the clinical failure of istiratumab in pancreatic cancer remain unclear and are subject to further analysis. This result, however, emphasizes the need for careful and detailed clinical exploration of the impact of potential biomarkers in each indication, including cancer cell related and tumor microenvironment mediated markers. In this respect, the relative role of other RTKs needs to be critically considered. In regard to ovarian cancer and ErbB3 for example, the randomized phase 2 trial of the anti-ErbB3 molecule seribantumab in combination with paclitaxel in patients with advanced platinum-resistant or refractory ovarian cancer showed an increased treatment benefit for patients whose tumors had detectable HRG mRNA and low HER2^9^.

In summary, our results suggest that the interplay between IGF-1R and ErbB3 may serve as a regulator of tumor growth and resistance to chemotherapies in ovarian cancer. Inhibition of both pathways may sensitize ovarian cancer tumors to chemotherapies representing an attractive therapeutic approach in selected patient populations.

## Methods

### Cell lines and reagents

OV90 cells were obtained from the American Type Culture Collection, OVCAR3, OVCAR5, and OVCAR8 cells were obtained from the National Cancer Institute, and PEA1, PEA2, PEO1, PEO4, PEO14, and PEO23 cells were obtained from the European Collection of Authenticated Cell Cultures. Cell lines were confirmed negative for mycoplasma prior to use, maintained according to manufacturer recommendations, and propagated for less than 8 weeks after initial plating. IGF-1 and HRG were obtained from R&D Systems. Istiratumab was produced by Merrimack Pharmaceuticals, paclitaxel was purchased from LC labs, pegylated liposomal doxorubicin was purchased from SunPharma, and cisplatin was purchased from Sigma.

### Multi-cellular tumor spheroid growth assays

Cells were seeded into gel- free scaffold-type microsquare 96-well nano-culture plates (SCIVAX) in 4% FBS, grown for 24 hours to allow for the formation of three-dimensional cell spheroids, then treated as indicated in the figure legends. Cell proliferation was assessed via Cell Titer-Glo® Luminescent Cell Viability Assay kit (Promega) as per the manufacturer’s instruction and luminescence was measured on a Synergy™ H1 plate reader.

### Quantitative flow cytometry

Cell surface receptor levels were quantified by flow cytometry as previously described^36^. Quantum Simply Cellular IgG bead standards (Bangs Laboratories) along with Alexa 647- or allophycocyanin (APC)-conjugated antibodies to quantify the number of surface receptors per cell. Beads and cells were analyzed using a FACSCanto™ system (BD Biosciences). Data were analyzed using FlowJo software (version 8.2).

### *In vitro* signaling experiments

For ELISA, cells were seeded into 96-well tissue culture plates (Costar) at 30,000 cells/well in media supplemented with 4% FBS. The following day, cells were synchronized by 24-hour serum starvation in media with 0% FBS. For western blot, cells were seeded into 10 cm dishes in 10% FBS media and allowed to grow for 24 hours before replacing the media with 0.5% FBS media. Signaling experiments were stopped with a cold PBS wash, and cell lysates were generated with Mammalian Protein Extraction Reagent (Thermo Scientific) supplemented with phosphatase and protease inhibitor pellets (Roche) and 150 mM sodium chloride (Sigma).

### Enzyme-linked immunosorbent assay (ELISA)

ELISAs were performed as previously described^12^. Briefly, high-binding assay plates (Corning) were coated with capture antibodies and incubated overnight followed by blocking with 2% bovine serum albumin (BSA, Sigma) in PBS for 1 hour. Plates were incubated with lysate diluted two-fold in 2% BSA, 0.1% Tween-20 PBS for 2 hours, then with primary detection antibodies for 2 hours, followed by secondary detection antibodies for 30 minutes. Chemiluminescent substrate (Pierce) was added to each plate for 20 minutes and luminescence measured using a Synergy™ H1 plate reader. Plates were washed 4 times with a PBS solution containing 0.05% Tween-20 between each incubation, and all incubations were done at room temperature.

### Western blot analysis

Samples were analyzed by western blotting as previously described^12^. Briefly, clarified cell lysates were boiled in LDS sample buffer (Life Technologies) at 95 °C for five minutes, and resolved by electrophoresis on 4–12% gels (Bio-Rad) using MES running buffer (Bio-Rad). Proteins were transferred to nitrocellulose membranes (Life Technologies) using an iBlot® device (Life Technologies) and membranes were blocked in blocking buffer (LI-COR Biosciences) for 1 hour at room temperature. Membranes were probed with primary antibodies (Cell Signaling Technology) in 5% BSA (Sigma), 0.1% Tween-20 tris-buffered saline solution (TBS-T) overnight at 4–8 degrees Celsius, washed 3 times for 10 minutes in TBS-T, followed by incubation with an anti-rabbit secondary antibody (Licor) in 5% milk (Cell Signaling Technologies) TBS-T for 45 minutes. After 3 additional 5-minute washes in TBS-T, bands were visualized on a LI-COR ODYSSEY® CLx imager. Protein bands were quantified using Image Studio (version 3.1.4) software.

### Cell line derived xenograft efficacy studies

All animal studies were performed according to the guidelines and with approval of the Institutional Animal Care and Use Committee at Merrimack Pharmaceuticals. Female athymic nude mice were obtained from Charles River Laboratories and were housed in a pathogen-free environment under controlled conditions and received food and water ad libitum. Tumors were established by subcutaneous injection of 5 × 10^6^ cells, suspended in 200 μL of 1:1 growth factor reduced Matrigel™ (Corning): unsupplemented culture media, into one shaven flank of recipient mice. Once the average measured tumor volume (calculated according to the formula: π/6 × (length × width × width)) had reached ~230 mm3, mice were randomized into groups and treatment was administered as outlined in figure legends. The average starting tumor volume per group was equivalent across all groups. Tumor volumes were measured twice weekly.

### Histology of tumor tissue

Ovarian tumor samples were commercially sourced from Avaden Biosciences, and assayed as previously described^13^. Briefly, tumor tissue samples were formalin fixed, paraffin-embedded, sectioned to 4–5 μm thickness, and analyzed using the Leica Bond Rx or Ventana Benchmark Discovery Platforms. Antibodies used for immunohistochemistry (IHC) were as follows: anti-IGF-1R (Ventana, G11), anti-ErbB3 (Cell Signaling Technology, D22C5). All IHC-stained specimens were scored by a board certified surgical clinical pathologist utilizing the clinical HER2 scoring system that classifies each sample into scores 0–3. Detection of IGF-1 (#313037), IGF-2 (#594367) and HRG (#311187) transcripts was performed using the *in situ* hybridization RNAscope® automated assay for the Leica Bond Rx (#321100) in accordance with protocols provided by Advanced Cell Diagnostics. For each tissue specimen, positive (PPIB, #313907) and negative (DapB, #312037) control RNA probes were evaluated to assess tissue quality and assay performance alongside scoring of each individual target probe, and scoring was in accoracnece with a 5 tiered scoring system (score 0–4) based on counting dots per cell. RNA quantity was scored based on manual counting described as follows. Staining results were categorized into five grades according to the number of dots visualized under a bright-field microscope. 0: No staining or less than 1 dot to every 10 cells (40X magnification); 1+: 1–3 dots/cell (visible at 20–40X magnification); 2+: 4–10 dots/cell, very few dot clusters (visible at 20–40X magnification); 3+: >10 dots/cell, and less than 10% positive cells have dot clusters (visible at 20X magnification); and 4+: >10 dots/cell, and more than 10% positive cells have dot clusters (visible at 20X magnification).

### Statistical analysis

Statistical significance, area under the curved calculations, and curve fitting analysis was performed using GraphPad Prism 7 software (version 7.03) as indicated in the figure legends.

## Supplementary information

Supplementary Information
