# Supplementary material for: Dual targeting of IGF-1R and ErbB3 as a potential therapeutic regimen for ovarian cancer
Source: Sci Rep. 2019 Nov 14;9:16832. doi: 10.1038/s41598-019-53322-y (PMC6856132; doi:10.1038/s41598-019-53322-y)
Supplement: Supplementary file 1 — Supplementary Information [file 41598_2019_53322_MOESM1_ESM.pdf]

Dual targeting of IGF-1R and ErbB3 as a potential therapeutic regimen for ovarian cancer

[1]Adam J. Camblin*,#, [1]Gege Tan*, [1]Michael D. Curley*, [1]Isabel Yannatos, [1]Sergio Iadevaia, [1]Victoria Rimkunas, [2]Mari Mino-Kenudson, [1]Troy Bloom, [1]Birgit Schoeberl, [1]Daryl C. Drummond, [1]Alexey A. Lugovskoy, [1]Chrystal U. Louis, [1]Vasileios Askoxylakis[#]

[1]Merrimack Pharmaceuticals, Inc., Cambridge, MA

[2]Massachusetts General Hospital, Harvard Medical School, Boston, MA

*These authors contributed equally to this work

Corresponding authors:

Vasileios Askoxylakis, M.D.
Merrimack Pharmaceuticals, Inc.
One Kendall Square
Cambridge, MA 02139
Phone: 617-441-1000
E-mail address: v.askoxylakis@gmail.com

Adam Camblin, PhD
Merrimack Pharmaceuticals, Inc.
One Kendall Square
Cambridge, MA 02139
Phone: 617-441-1000
E-mail address: adam.camblin@gmail.com

Subject terms: IGF1R, ErbB3

Conflict of interest statement: All authors affiliated with Merrimack Pharmaceuticals were employees of Merrimack Pharmaceuticals at the time of the study, with only AJC, IY, and DCD currently owning stock or stock options in Merrimack Pharmaceuticals.

Data will be made available upon request.

Authorship contribution: AJC, GT, MDC, IY, VR, MMK, and TB contributed to the generation of data. AJC, GT, MC, SI, and VR contributed to the analysis of data. MDC, BS, DCD, AAL, CUL, and VA conceived of the work and supervised its execution. AJC and VA wrote the manuscript. All authors have reviewed the manuscript and approve of its publication.

Supplemental Figure Legends

Figure S1: IGF-1R expression correlated with reduced sensitivity to chemotherapy in ovarian cancer cell lines. A, Cell surface expression of EGFR, HER2, and cMET were assessed via quantitative flow cytometry across the fifteen ovarian cancer cell lines. B, Cell line responsiveness to paclitaxel or cisplatin from Figure 1A correlated to cell surface receptor expression from Figure S1A. C, Weighted results from PLSR analysis correlating receptor expression with chemotherapy sensitivity.

Figure S2: Induction of AKT phosphorylation in response to growth factor stimulation across ovarian cancer cell line panel. Cell lines were serum-starved for 24 hours and then treated for 15 minutes with 100 ng/mL of each growth factor. Cell lysates were collected, and pAKT S473 was determined by ELISA. Levels for ng/mL pAKT were control-subtracted and maximum-normalized within each cell line.

Figure S3: Basal expression of growth factor mRNA across panel of ovarian cancer cell lines. Cell lines were grown in compete media before being lysed for mRNA isolation using the RNeasy kit from Qiagen. mRNA expression levels for 8 different growth factors was assessed by RTRTPCR, followed by normalization to housekeeping genes using the delta-deltaCt method. mRNA expression was maximum normalized within each growth factor.

Figure S4: Receptor expression levels do not correlate with sensitivity to istiratumab monotherapy. qFACS cell surface receptor expression levels from figure 1 are plotted against istiratumab sensitivity from figure 3. No statistically significant correlation is observed.

Figure S5: Effects of chemotherapy alone on AKT phosphorylation. OVCAR8 cells were treated as described in figure 5. No statically significant changes were observed in AKT phosphorylation following monotherapy chemotherapy treatment under these conditions.

Figure S1: cMET expression correlates with reduced sensitivity to chemotherapy in ovarian cancer cell lines

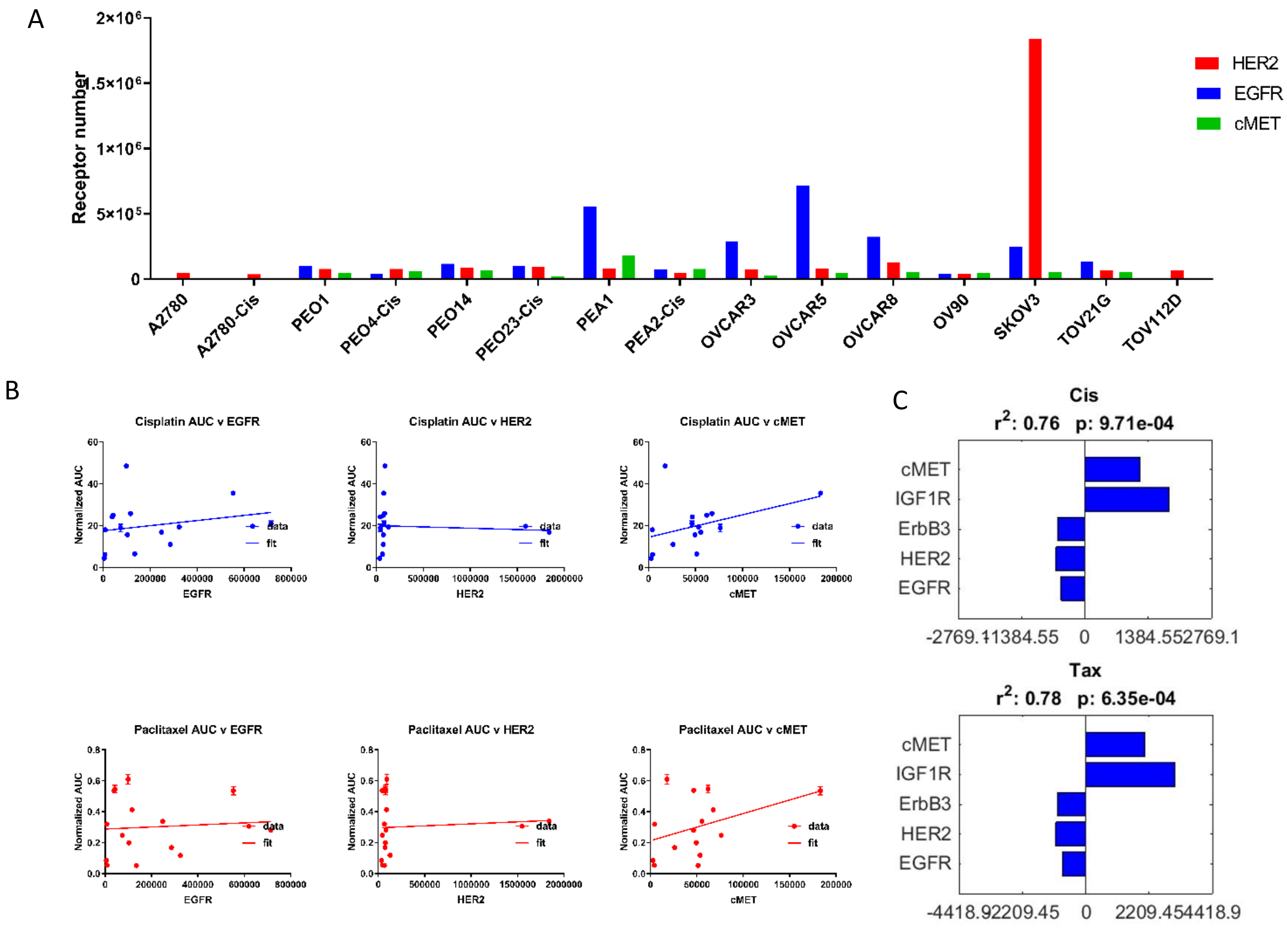

Figure S2: Induction of AKT phosphorylation in response to growth factor stimulation across ovarian cancer cell line panel

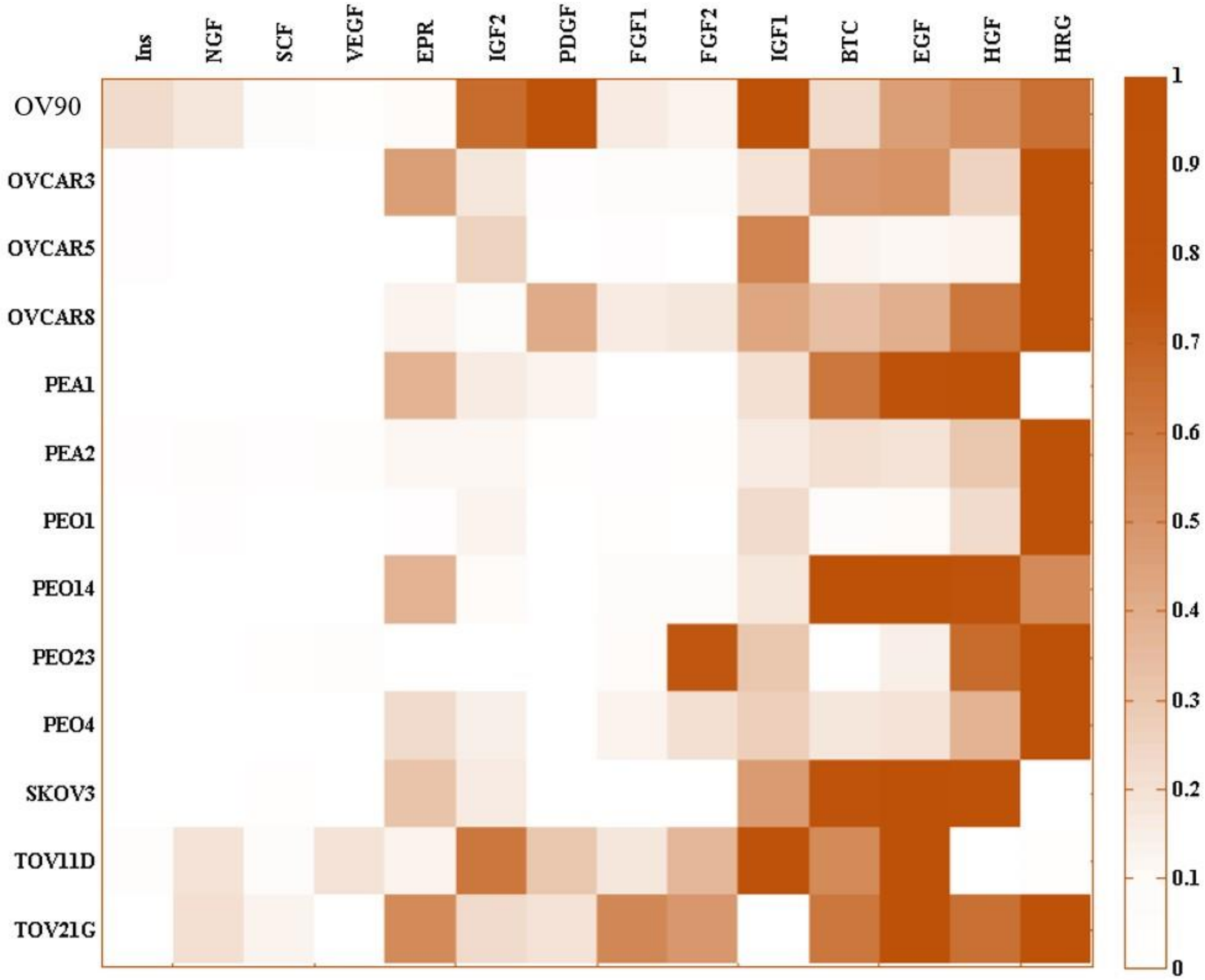

Figure S3: Basal expression of growth factor mRNA across panel of ovarian cancer cell lines.

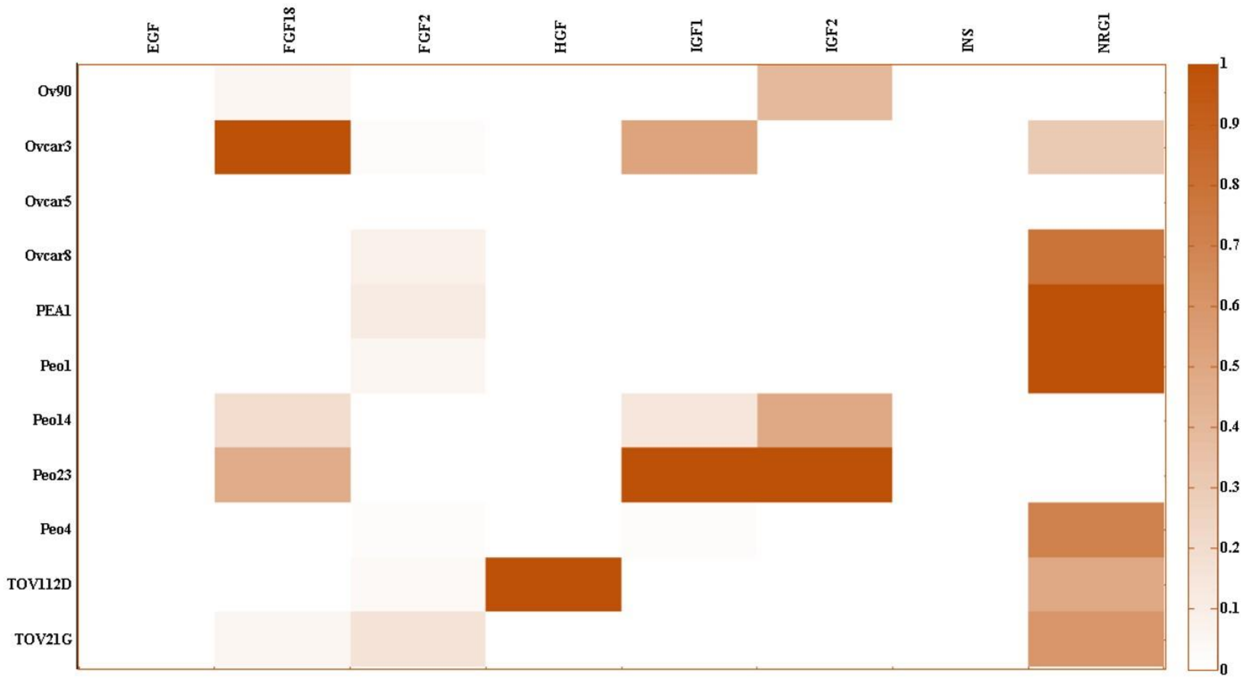

Figure S4: Receptor expression levels do not correlate with sensitivity to istiratumab monotherapy

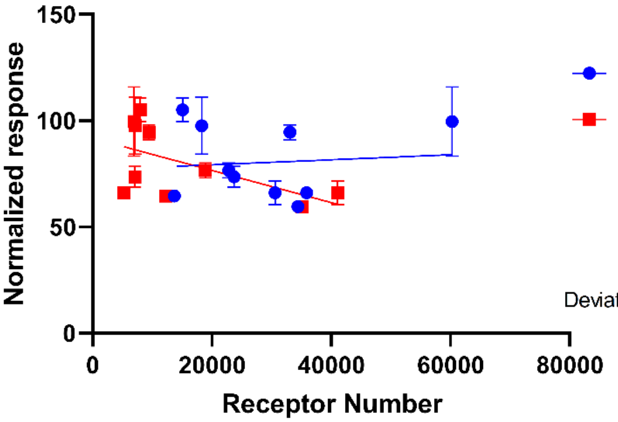

	IGF1R	ErbB3
R square	0.008965	0.3197
P value	0.7947	0.0885
Deviation from zero?	Not Significant	Not Significant

Figure S5: Chemotherapy alone does not increase pAKT with statistical signficicance

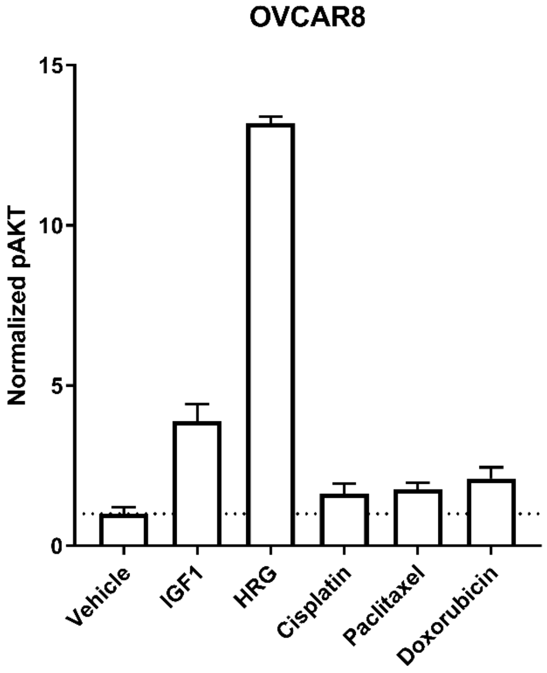

Dunnett's multiple comparisons test	Mean Diff.	95.00% CI of diff.	Significant?	Summary	Adjusted P Value
Vehicle vs. Cisplatin	-0.6333	-2.396 to 1.129	No	ns	0.2942
Vehicle vs.Paclitaxel	-0.7667	-1.648 to 0.1146	No	ns	0.0651
Vehicle vs.Doxorubicin	-1.100	-2.953 to 0.7530	No	ns	0.1306

Table S1: Patient expression of growth factor or mRNA expression levels presented in figure 2A as percentages of total population.

	0	1+	2+	3+	4+
HRG	55	45			
IGF-2	9	18	0	18	55
IGF-1	0	73	9	18	
ErbB3	0	8	58	34	
IGF1R	0	0	27	73	
